# Psychedelic science in post-COVID-19 psychiatry

**DOI:** 10.1017/ipm.2020.94

**Published:** 2020-08-19

**Authors:** J. R. Kelly, M. T. Crockett, L. Alexander, M. Haran, A. Baker, L. Burke, C. Brennan, V. O’Keane

**Affiliations:** 1 Department of Psychiatry, Trinity College Dublin, Dublin, Ireland; 2 Tallaght University Hospital, Dublin, Ireland; 3 Department of Neurological Intervention and Imaging, Sir Charles Gairdner Hospital, Perth, Western Australia, Australia; 4 Health Service Executive, Dublin, Ireland; 5 Daughters of Charity Disability Services, Dublin, Ireland; 6 Sheaf House, Exchange Hall, Tallaght, Dublin, Ireland

**Keywords:** Coronavirus, COVID-19, depression, psilocybin, psychedelics, selective serotonin reuptake inhibitors, treatment-resistant depression

## Abstract

The medium- to long-term consequences of COVID-19 are not yet known, though an increase in mental health problems are predicted. Multidisciplinary strategies across socio-economic and psychological levels may be needed to mitigate the mental health burden of COVID-19. Preliminary evidence from the rapidly progressing field of psychedelic science shows that psilocybin therapy offers a promising transdiagnostic treatment strategy for a range of disorders with restricted and maladaptive habitual patterns of cognition and behaviour, notably depression, addiction and obsessive compulsive disorder. The COMPASS Pathways (COMPASS) phase 2b double-blind trial of psilocybin therapy in antidepressant-free, treatment-resistant depression (TRD) is underway to determine the safety, efficacy and optimal dose of psilocybin. Results from the Imperial College London Psilodep-RCT comparing the efficacy and mechanisms of action of psilocybin therapy to the selective serotonin reuptake inhibitor (SSRI) escitalopram will soon be published. However, the efficacy and safety of psilocybin therapy in conjunction with SSRIs in TRD is not yet known. An additional COMPASS study, with a centre in Dublin, will begin to address this question, with potential implications for the future delivery of psilocybin therapy. While at a relatively early stage of clinical development, and notwithstanding the immense challenges of COVID-19, psilocybin therapy has the potential to play an important therapeutic role for various psychiatric disorders in post-COVID-19 clinical psychiatry.

Crises induce a wide range of psychological reactions, with varying degrees of adaptability. The combination of uncertainty and social distancing induced by the COVID-19 pandemic can lead to excessive fear/anxiety, loneliness and depressive thoughts (Holmes *et al.* 2020, Luykx *et al.* 2020, Vindegaard & Benros, 2020). While the medium- to long-term mental health consequences are not yet known, an increase in psychological and psychiatric problems are predicted (Horesh & Brown, 2020, O’Connor *et al.* 2020, Türközer & Öngür, 2020), with an excess burden on vulnerable groups (Kelly, 2020). The implementation of a range of multidisciplinary strategies across socio-economic and psychological levels may be needed to mitigate the mental health burden of COVID-19.

Accumulating clinical data shows that psilocybin therapy may be an effective therapeutic strategy across a range of disorders, including depression (Carhart-Harris *et al.* 2016, Davis *et al.* 2019), obsessive compulsive disorder (Moreno *et al.* 2006) and addiction disorders (Garcia-Romeu *et al.* 2019, Johnson *et al.* 2017). In addition, clinical trials are underway to investigate psilocybin therapy in anorexia nervosa (NCT04052568) and there may be a role for psilocybin therapy in the treatment of anxiety disorders (Weston *et al.* 2020).

Recent advances in psychedelic science are gradually unravelling the multimodal mechanisms underlying the therapeutic effect of psilocybin therapy (for example Carhart-Harris & Friston, 2019, Lord *et al.* 2019, Preller *et al.* 2020, Varley *et al.* 2020). Psilocybin reliably alters an individual’s state of consciousness, probably through agonist mechanisms at the 5-HT2A receptor, especially in the deep pyramidal cells in the cortex (Nutt *et al.* 2020). The transient, dose-dependent alteration of the complex interconnected neural networks of the brain (Lord *et al.* 2019, Varley *et al.* 2020) encompassing the self-reflecting ‘ego’, induced by psilocybin, can lead to profound experiences of connectivity to others and the environment (Erritzoe *et al.* 2018, Griffiths *et al.* 2006, 2016, Grob *et al.* 2011, Kettner *et al.* 2019, Smigielski *et al.* 2019) and can be harnessed by psilocybin therapy to re-conceptualise restricted and maladaptive habitual patterns of cognition and behaviour.

As such, psilocybin therapy provides a translatable, transdiagnostic treatment strategy that can be further refined by a precise-personalised approach (Kelly *et al.* 2017, Lewis *et al.* 2020, Preller *et al.* 2016, 2020, Studerus *et al.* 2012). Advancing precise-personalised psilocybin therapy is of particular importance given the individual variation in responses, high rates of relapse in psychiatric disorders and contraindication in psychotic and manic conditions (Carhart-Harris *et al.* 2018). It has been suggested that internalising disorders may be a useful broad construct for the therapeutic application of psilocybin therapy (Nutt & Carhart-Harris, 2020). Moreover, given the transdiagnostic potential, a dimensional framework (Insel, 2014) that aligns with bio-psycho signatures could also be leveraged to enhance the targeted application of psilocybin therapy and further unravel the mechanisms underpinning the acute and persistent therapeutic effects. Indeed, further exploration of psilocybin’s impact on neuroimmunoendocrine pathways (Galvão *et al.* 2018, Hasler *et al.* 2004, Nau *et al.* 2013, Strajhar *et al.* 2016, Szabo, 2015), including the microbiome–gut–brain axis, may provide additional insights into the persisting therapeutic effects (Kelly *et al.* 2019c, Kuypers, 2019).

Notwithstanding the limitations of animal models in fully capturing the different aspects of psilocybin therapy (Jefsen *et al.* 2019, Meinhardt *et al.* 2020), preclinical data have shown that serotonergic psychedelics, including psilocybin, can induce hippocampal neurogenesis (Catlow *et al.* 2013, Morales-Garcia *et al.* 2017, Vaidya *et al.* 1997), promote dendritic spine growth and stimulate synapse formation in the prefrontal cortex (González-Maeso *et al.* 2007, Ly *et al.* 2018). Preclinical data also suggest that psychedelics lead to 5-HT2A receptor-mediated glutamate release (Ly *et al.* 2018), and a recent magnetic resonance spectroscopy study in healthy humans found that psilocybin administration was associated with increased glutamate in the medial prefrontal cortex (Mason *et al.* 2020).

Researchers from the Center for Psychedelic and Consciousness Research at Johns Hopkins University recently focussed on the claustrum, a thin sheet of grey matter, embedded in the white matter of the cerebral hemispheres and situated between the putamen and the insular cortex, with a rich supply of 5-HT2A receptors and glutamatergic connectivity to the cerebral cortex, and thought to be associated with cognitive task switching (Barrett *et al.* 2020b, Krimmel *et al.* 2019). Psilocybin acutely reduced claustrum activity and altered its connectivity with the default mode network and frontoparietal task control network, in a study involving 15 healthy volunteers, thus implicating this region as a key mediator in psilocybin therapy (Barrett *et al.* 2020b).

The same research group, in an open-label pilot study of 12 healthy volunteers, showed that psilocybin reduced both negative affect and amygdala responses to emotional stimuli 1 week after psilocybin, whereas by 1 month after psilocybin the responses returned to baseline (Barrett *et al.* 2020a). At both 1 week and 1 month after psilocybin, there were global increases in brain functional connectivity (Barrett *et al.* 2020a). A previous study in healthy controls also showed reduced amygdala reactivity, particularly on the right side, to negative and neutral stimuli due to psilocybin (Kraehenmann *et al.* 2015). In contrast, an open-label study of 19 subjects with treatment-resistant depression (TRD) showed that psilocybin increased amygdala responses to emotional faces (Roseman *et al.* 2018) and decreased functional connectivity between the ventromedial prefrontal cortex and the right amygdala 1 day after psilocybin (Mertens *et al.* 2020). Larger studies may be needed to resolve the complexities.

In the midst of this evolving ‘Psychedelic Revolution in Psychiatry’ (Nutt *et al.* 2020) and potential increasing recreational psychedelic use, albeit from 0.55% in 2015 to 0.86% in 2018, in a sample of 168,000 members of the public (Yockey *et al.* 2020), the Royal Australian and New Zealand College of Psychiatrists (RANZCP) recently published a clinical memorandum on the ‘Therapeutic use of psychedelic substances’ (RANZCP, 2020). This memorandum acknowledges not only the emerging therapeutic potential of psychedelics but also the need for more efficacy and safety data, particularly on potential long-term effects, to inform future potential use in psychiatric practice.

In terms of acceptability and tolerability, results from the Global Drug Survey (2019) of 85,000 people showed only 18% of those surveyed, who have never used psychedelics, said they would accept psilocybin therapy for depression or PTSD, increasing to 59% in those who had previously tried psychedelics (Winstock & Johnson, 2019). The reported fears related to ‘brain damage and bad trips’ (Winstock & Johnson, 2019). Psilocybin therapy data from John Hopkins University, over a 16-year period, encompassing 250 volunteers and 380 sessions, reported no major psychological issues, with 0.9% of volunteers experiencing minor and transient psychological issues (Carbonaro *et al.* 2016). However, high-quality clinical data on the long-term effects of psychedelics are lacking. For example, there is very limited data on hallucinogen-persisting perception disorder (HPPD), a rare condition that involves the continued presence of sensory disturbances (Halpern *et al.* 2018; Martinotti *et al.* 2018; Orsolini *et al.* 2017). A review by Halpern and colleagues suggests that HPPD is, in most cases, due to a ‘subtle over-activation of predominantly neural visual pathways that worsens anxiety after ingestion of arousal-altering drugs, including non-hallucinogenic substances’ (Halpern *et al.* 2018). The authors note that a personal or family history of anxiety and pre-drug use complaints of tinnitus, eye floaters and concentration problems may predict vulnerability for HPPD (Halpern *et al.* 2018). Similarly, the impact of regular psychedelic use on the brain is limited (Bouso *et al.* 2015; Halpern *et al.* 2005). Although, it is important to note that psilocybin therapy studies do not use regular dosing, using between 1 and at most 3 doses of psilocybin.

Dublin is one of the clinical trial centres participating in a double-blind randomised controlled phase 2b COMPASS trial of psilocybin therapy in TRD (COMP001) (Kelly *et al.* 2019a). Results from this large scale trial, and others, will address concerns regarding psilocybin safety, efficacy and dose optimisation. Moreover, we eagerly await the results from the potentially paradigm shifting, double-blind trial of psilocybin therapy *versus* the selective serotonin reuptake inhibitor (SSRI) escitalopram in depression from the Centre for Psychedelic Research at Imperial College London (Psilodep-RCT, NCT03429075) (Nutt & Carhart-Harris, 2020) and acknowledge that for some people with depression, SSRIs and psilocybin may become ‘competitive options’ despite postulated mechanistic complementarity, with SSRIs enhancing 5-HT1AR pathway and psilocybin enhancing the 5-HT2AR pathway (Carhart-Harris & Nutt, 2017). However, many people with depression may choose to remain on antidepressants (Kelly *et al.* 2019b) and it is important to determine the safety and efficacy of this approach. 5-HT2AR antagonists, such as ketanserin, block the therapeutic effect of psilocybin (Preller *et al.* 2017), whereas the partial 5-HT1A agonist buspirone may exert inhibitory effects (Pokorny *et al.* 2016). However, apart from anecdotal evidence suggesting a blunted effect (Bonson *et al.* 1996; Bonson & Murphy, 1996), psilocybin therapy in conjunction with SSRI’s has never been investigated in TRD.

The gradual emergence from COVID-19 lockdown will see the launch of a new COMPASS clinical study (COMP003) in Dublin and San Diego to determine the antidepressant effect of psilocybin therapy in people with TRD who continue SSRI medication. This exploratory open-label trial will aim to recruit 20 participants with a single or recurrent episode of at least moderate clinical depression between 3 months and 2 years duration that has not responded to an adequate dose and duration of at least two pharmacological treatments. A single dose of oral psilocybin of 25mg will be administered with psychological support to participants who have been taking an SSRI’s for at least 6 weeks. The results of this study could have important practical implications for the future of psilocybin therapy and may have implications for future phase 3 trials in TRD, which could pave the way for the integration of psilocybin therapy into clinical psychiatry.

However, both clinical and research psychiatry have been transformed by COVID-19, demanding additional strategies to overcome the considerable challenges (O’Brien & McNicholas, 2020; Türközer & Öngür, 2020). To mitigate the spread of COVID-19 and facilitate the safe reopening and progress of ongoing psilocybin trials, in line with local and national guidelines, a number of measures will be implemented. These include, for example, participant and researcher respiratory symptom checklists, regular temperature checks, access to COVID-19 testing (if indicated), meticulous attention to extra hygiene measures, personal protective equipment (where not expected to negatively impact the participant’s experience), and the option of remote study visits (where possible by the protocol). Notwithstanding the challenges and the early stage of clinical development, psilocybin therapy, at the forefront of translational neuroscience and psychiatry, is likely to play an important therapeutic role for certain conditions in post-COVID-19 clinical psychiatry.
